# Knowledge, Experience and Perception of Gender-Based Violence Health Services: A Mixed Methods Study on Adolescent Girls and Young Women in Tanzania

**DOI:** 10.3390/ijerph18168575

**Published:** 2021-08-13

**Authors:** Caroline Mtaita, Samuel Likindikoki, Maureen McGowan, Rose Mpembeni, Elvis Safary, Albrecht Jahn

**Affiliations:** 1 Heidelberg Institute of Global Health, Im Neuenheimer Feld 130.3, 69120 Heidelberg, Germany; maureen.mcgowan@uni-heidelberg.de (M.M.); elvis.safary@uni-heidelberg.de (E.S.); albrecht.jahn@uni-heidelberg.de (A.J.); 2 Department of Psychiatry and Mental Health, School of Medicine, Muhimbili University of Health and Allied Sciences, Dar es Salaam 65001, Tanzania; likindikoki@gmail.com; 3 Department of Epidemiology, School of Public Health and Social Sciences, Muhimbili University of Health and Allied Sciences, Dar es Salaam 65015, Tanzania; rcmpembeni@gmail.com

**Keywords:** gender-based violence, knowledge, adolescent girls, young women, Tanzania

## Abstract

Many adolescent girls and young women (AGYW) experience gender-based violence (GBV) in Tanzania and only few seek GBV health services following violence. The objectives of our study are (1) to evaluate knowledge of gender-based violence among AGYW, (2) to explore their perceptions of and experiences with GBV health service quality and (3) to evaluate access to comprehensive GBV services. This study employed an explanatory, sequential mixed methods design in two districts of Dar es Salaam, Tanzania (Kinondoni and Temeke). A quantitative cross-sectional survey among AGYW (n = 403) between 15–24 years old was performed to assess their knowledge of GBV as well as perceptions of and experiences with GBV health services. The quantitative data was complemented by 20 semi-structured in-depth interviews with participants. Out of 403 study participants, more than three quarters (77.9%) had moderate to good knowledge of how GBV is defined and what constitutes gender-based violence. However, few participants (30.7%, n = 124) demonstrated knowledge of GBV health services offered at local health facilities. For example, only 10.7% (n = 43) of participants reported knowledge of forensic evidence collection. Additionally, of 374 participants (93% of total participants) who reported to have received GBV education sessions, only 66% accessed GBV health services (n = 247) and about half of these (52.7%, n = 130) were satisfied with these services. The study indicated that—despite good knowledge about what constituted GBV—knowledge about the roles and availability of GBV health services was limited and utilization of GBV health services remained low. Coordinated actions need to be strengthened to reach AGYW who remain unaware of GBV health services offered at health facilities by improving GBV service quality, bettering interventions aimed at reducing GBV among AGYW in Tanzania, and scaling-up integrated service models, such as GBV one-stop centers.

## 1. Introduction

Violence against women is a major impediment to the fulfillment of women’s rights and to the achievement of the sustainable development goals (SDGs) [1]. The World Health Organization (WHO) defines violence as the intentional use of physical force or power (threatened or actual) against oneself, another person or a group/community that results in, or has a high likelihood of resulting in injuries, death, psychological harm or deprivation [2,3]. The risk of being subjected to gender-based violence (GBV) is prominent among women and girls, particularly those belonging to ethnic minorities, those of low socioeconomic status and those who are poorly educated resulting in severe social, economic, physical and mental health consequences [4,5,6]. The term gender-based violence enables one to understand the intersection between different forms of violence and gender [7]. A sociological theory of gender-based violence as used in our study further defines these intersections as existing within micro (e.g., one’s own understanding of gender roles and norms), meso (e.g., interpersonal relationships consistent with gender norms) and macro (e.g., systematic gender constructs) levels [8]. 

According to a WHO 2013 report, nearly one-third of women globally aged 15 years and above have experienced physical and/or sexual violence with the highest instances of GBV reported in Africa and Asia [9]. A WHO study on violence and women’s health across 10 countries including Tanzania, reported that between 13–62% of women had experienced physical violence over the course of their lifetime, 29% reported violence within the past year, while only 3% had sought GBV health services [10]. Additionally, the report demonstrated that GBV particularly in African countries was a major cause of ill health among women and girls, as it can cause disability due to injuries, a range of physical and mental impairments and can even result in death [11]. A study conducted in Kenya aiming to explore the uptake of GBV services, found that the majority GBV survivors had no knowledge of available GBV recovery services (GBVR), the benefits of treatment, nor their need for treatment. There were also limited records of service utilization despite GBVR being free of charge [12]. Similarly, a study conducted in the Democratic Republic of Congo (DRC) revealed that 85% of women reported being victims of sexual violence while nearly half (45%) reported never having received GBV health services [13].

Access to GBV health services among adolescent girls and young women (AGYW) is a growing body of concern [14]. Several studies have reported that health care seeking behavior is a multifarious, reflecting socio-demographics and health characteristics which can thereby challenge access to GBV services [15,16,17]. Increasing importance is also being given to aspects of GBV (e.g., impact of violence, its consequences and nature of violence) and its health service response [18,19,20,21,22]. Health systems play a critical role in supporting women, minimizing the impact of violence and preventing future violence. Health practitioners are often the first responders for abused women and when health care workers (HCWs) can identify and treat victims of violence, they represent a valid opportunity to direct women to communities and services that can provide legal assistance [23,24]. However, there is rampant underutilization of GBV health services in sub-Saharan Africa countries with some countries having no record of accessed services at all [25].

As it is in other parts of the world, Tanzania is no exception to GBV and many forms of violence are in fact considered socially acceptable [26]. The vast majority of AGYW in Tanzania experience violence in their homes by someone familiar to them (partner/husband, family member, friend or neighbor) [26]. As per the 2016 Tanzania Demographic Health Survey (TDHS 2016), 43.6% of women aged 15–49 reported experiencing lifetime physical and/or sexual violence by their partner, husband or stranger. Additionally, sexual violence among AGYW was shown to be as high as 46% in Dar es Salaam [27]

The number of women who seek GBV health services in Tanzania remains low [27]. The TDHS 2016 indicates that less than 1.1% of women aged 15–49 years who experienced physical and sexual violence sought GBV health care services [27]. Furthermore, a study conducted in rural Tanzania (Morogoro district), revealed that women who had only completed primary school education had lesser GBV knowledge compared to those who had secondary school education- thereby highlighting additional barriers to GBV service access [28]. Considering these challenges, the Government of Tanzania in collaboration with the Ministry of Health, Community Development, Gender, Elderly and Children (MoHCDGEC) and other stakeholders have gone to great lengths to strengthen the health service response for GBV survivors. For example, the development of the National Plan of Action for the Prevention and Eradication of Violence against Women and Children (NPA-VAWC) 2017/18–2021/22 highlights the importance of efficient and effective police response, gender-sensitive prosecution services, as well as health and social welfare services to address violence against women and children. For example, one-stop centers have been established that provide medical, legal and psycho-social services for GBV survivors under one roof [27]. The strategy further supports the provision of easily accessible information on GBV services available to women and children [27]. Thus, special attention has been given by the Government of Tanzania to train and motivate health care workers to make GBV health services accessible, acceptable, appropriate and more user-friendly to victims of GBV.

Despite government initiatives and programs to facilitate utilization of GBV services in Tanzania, there is evidence that barriers persist. For example, a study indicated that women were more likely to seek health services when violence was perpetrated by a stranger—thereby posing challenges seeking health services when violence was committed by someone familiar to the victim [26]. Additionally, GBV within sex work poses major challenges to women reporting instances of GBV and visiting health facilities [29]. Even though some barriers to GBV services access have been identified, little is known about the influence of GBV knowledge and prior GBV service experiences on GBV service access.

This study aimed to better understand the knowledge of gender-based violence among adolescent girls and young women and to analyze their perceptions of and experiences with GBV health services in terms of access and quality. This research will inform the design of future GBV prevention interventions to support current victims of violence and to prevent future instances of violence.

## 2. Materials and Methods

### 2.1. Study Setting

The study sites were in Temeke and Kinondoni districts of Dar es Salaam. Dar es Salaam is the largest city in Tanzania and comprises of five districts (Temeke, Kinondoni, Ilala, Ubungo and Kigamboni). Temeke and Kinondoni districts were selected for this study because research conducted in Tanzania found that AGYW in these districts reported the highest incidences of GBV [30].

### 2.2. Study Design and Population

The study utilized a mixed method, explanatory sequential design where quantitative data was first collected followed by qualitative data [31]. This study was part of a larger ongoing community-based project (John Hopkins Program for International Education in Gynecology and Obstetrics, JHPIEGO SAUTI), which was conducted among adolescent girls and young women 15–24 years old in Temeke and Kinondoni districts. The basis of this study was to explore AGYW knowledge of GBV as well as their perceptions of experience with GBV health services following violence. The study has been divided into two parts: part one indicating the quantitative study and part two indicating the qualitative study.

### 2.3. Study Context

This study functioned within an ongoing JHPIEGO SAUTI project, a comprehensive community outreach program that implements a peer-based HIV and GBV prevention intervention for AGYW in 14 regions of Tanzania. The project’s overarching aim is to reduce instances of HIV infections among vulnerable populations including AGYW, sex workers and people who inject drugs by providing biomedical (e.g., HIV testing, STI screening and family planning services) and structural interventions (e.g., GBV interventions and alcohol and drug screenings) at community level. The JHPIEGO SAUTI project identifies target groups through snowballing methods in hotspots including brothels, bars, markets, mining centers and truck shops.

In each target district within this study (Kinodoni and Temeke districts), JHPIEGO SAUTI partnered with civil society organizations (CSOs) working with the target population. Selected CSOs employed peer educators who were responsible for identifying AGYW and escorting them to a project representative for GBV service provision through traceable linkages to care, treatment or other referral services defined as “escorted referrals”. Further information about the JHPIEGO SAUTI project can be found under https://www.usaid.gov/documents/1860/sauti-project (accessed on 12 August 2021). 

#### 2.3.1. Inclusion Criteria

AGYW were eligible for study inclusion if they were 15–24 years old and enrolled in the JHPIEGO SAUTI project, self-reported experiencing at least one form of violence (physical, mental and/or sexual violence), resided in either Temeke or Kinondoni districts and voluntarily agreed to participate in the study.

#### 2.3.2. Exclusion Criteria

AGYW who were not enrolled in the ongoing JHPIEGO SAUTI project and those who were physically and/or mentally unfit to be interviewed (or unable to provide consent) were not included in the study.

### 2.4. Part one: Quantitative Study 

#### 2.4.1. Study Design

We conducted a cross-sectional survey on knowledge of GBV as well as perceptions of and experiences with GBV health services among a population of AGYW. Participants were selected by CSOs working in collaboration with the JHPIEGO SAUTI project.

#### 2.4.2. Sampling and Sample Size

**Figure 1:**
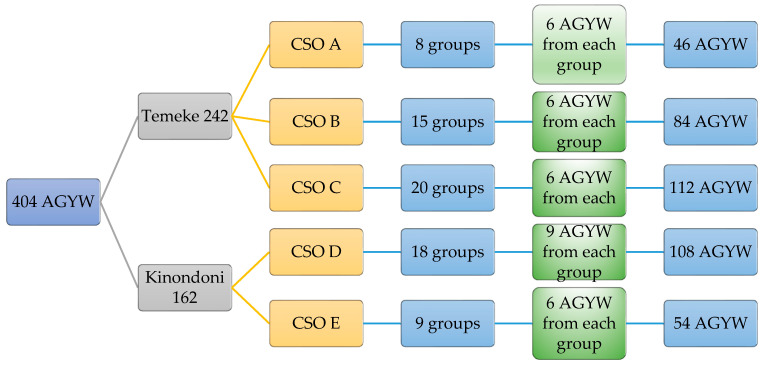
Proportionate sampling tree for AGYW participants.

In step one, a required sample size of 404 participants was calculated by a 40% proportion of GBV among AGYW in Dar es Salaam using a 95% confidence interval and 5% margin of error [32]. One questionnaire was incomplete and was therefore not included in analysis, hence a total of 403 respondents were recorded. In step two, proportionate sampling was used to calculate the number of participants required from each district which was determined by dividing the district CSOs by the total number of CSOs in the JHPIEGO SAUTI and multiplied by the required sample size ($\frac{District\;CSOs}{Total\;CSOs})\times 404$. A total of 162 participants were recruited from Kinondoni district and 242 participants from the Temeke district. In step three, we obtained two separate lists of all AGYWs, one list of AGYW who had received GBV services through JHPIEGO SAUTI and the second list of those who did not receive GBV health services. In step four, participants were randomly selected from each of these lists interchangeable until the desired number was reached. Figure 1 illustrates the sampling methods used in this study.

#### 2.4.3. Quantitative Data Collection

Participants were interviewed using an administered survey developed between researchers and JHPIEGO SAUTI. Data on socio-demographics (e.g., age, marital status, occupation, income and highest educational level attained), knowledge of GBV definitions, perceptions of and experiences with GBV health services including reasons for refusing GBV health referrals were obtained. The face-to-face survey took approximately 40–55 min to administer. This was used to obtain a deeper understanding on the topics. The research assistants received a one-day training delivered by the lead researcher on survey delivery techniques and the content of the survey.

#### 2.4.4. Data Analysis

Descriptive statistics (frequencies and proportions) were used to present the data. We operationalized our outcomes as follows: (a) knowledge of GBV definition. This was an open-ended question and correct answers were scored according to our interpretation of the GBV definition provided by the WHO [9]. According to our interpretation of the WHO definition of GBV, a correct definition of GBV captures the range of acts committed by a perpetrator, subjective experiences of a victim, consequences of harm and forms of violence. The results were scored as “good”, if a respondent mentioned all four parameters, “moderate” if a respondent mentioned at least two out of the four parameters, and “limited” if none or one parameter was mentioned; (b) perceptions of and experiences with GBV health services were measured on a five-point Likert scale (strongly agree, agree, neutral, disagree and strongly disagree). For statistical analyses, we categorized the Likert scale as “strongly agree/agree”, “neutral” (neither agree nor disagree) or “strongly disagree/disagree”; (c) access to GBV health services and (d) reasons for refusing GBV health services referral following GBV education services. Outcomes were defined and explained to the participants for ease of understanding. Quantitative data was collected and compiled using Microsoft Excel. We employed STATA 13.0 statistical software (StataCorp, College Station, TX, USA).

### 2.5. Part Two: Qualitative Study 

#### 2.5.1. Study Design

The qualitative element of this study utilized a narrative approach to conduct and analyze in-depth interviews by focusing on contextual meaning of the text obtained from the narrative responses as described by H.F. Hsieh and S.E. Shannon [33]. This approach gives the interviewee the opportunity to express her experiences and perceptions on a topic of interest while allowing the interviewer flexibility to explore new areas of interest. Here the interviewers collected data that fell into three thematic areas (a) knowledge about the definition of gender-based violence; (b) perceptions of and experience with GBV health services; and (c) reasons for refusing GBV health service referral.

#### 2.5.2. Sampling and Sample Size

This study purposively sampled 10 participants from each target district for a total of 20 in-depth interviews. Participants provided insights to their knowledge about GBV as well as their perceptions of and experiences with GBV health services.

#### 2.5.3. Qualitative Data Collection

The in-depth interviews (IDIs) were conducted in the local language (Kiswahili) with participants regarding their perceptions of and experiences with GBV health services and their understanding of what constitutes GBV. Interviewers probed during the interviews whenever necessary to enrich the data collected to obtain a deeper understanding of the participant’s perspectives [34]. All interviews were conducted in quiet and private places (e.g., homes or places within the community) chosen by the participants to ensure safety regarding the sensitive nature of this study. Each IDI took approximately 30–45 min to conduct.

#### 2.5.4. Qualitative Data Analysis

Researchers used preliminary data analysis to identify emergent themes. The IDIs were audio-recorded, transcribed verbatim and translated from Kiswahili into English by two independent Tanzanian researchers. Two authors read all of the transcripts multiple times for familiarization and deep understanding of the information. The data was indexed and grouped into themes for easy retrieval, review and further exploration. The transcripts were then exported into Microsoft Excel for thematic analysis. A thematic framework was drawn from the inductive codes which arose from the interviews. Inductive codes were subsequently added from GBV literature to address knowledge of GBV, perceptions of and experiences with GBV health services, as well as access to GBV health services. The transcripts and codes were reviewed and agreed upon via consensus by three independent researchers. The final step of the analysis was connecting interrelated themes to construct a narration [33]. Qualitative analyses were manually performed using Microsoft Word.

#### 2.5.5. Mixed Methods Integration

We triangulated quantitative results with results from qualitative thematic content analysis to frame our results. We specifically used a narrative approach to structure the identified themes in the qualitative study to guide our explanatory results. According to Fetters et al. (2013) [35], when similar conclusions are obtained from merged numeric and textual data, confirmation of findings provide greater credibility to the results.

### 2.6. Ethical Considerations

This study respected the principles of the Declaration of Helsinki. All methods performed in this study were in accordance with the ethical standards of the institutions and national research committees. The study was granted ethical approval by the Medical Research Coordinating Committee (MRCC) of the National Institute for Medical Research (NIMR) in Tanzania (NIMR/HQ/R.8a/Vol.IX/2986) and Ethics Committee of the Medical Faculty of Heidelberg University (S-737/2018). Approval to collect data was obtained through official permission from respective central and local government authorities and leaders. Permission to access the AGYW groups was granted by the JHPIEGO SAUTICountry Director. All participants provided written informed consent for participation in the study and for participants <18 years old, consent was obtained from their parent or guardian. Confidentiality of the participants is maintained in which no names or identifying information are used in the presentation of this research.

## 3. Results

We present our results according to three topic areas: (1) knowledge of gender-based violence among AGYW, (2) perceptions of and experiences with GBV health services among AGYW and (3) access to and refusal of GBV health services among AGYW. For each topic, key quantitative results are presented alongside qualitative findings.

### 3.1. Socio-Demographic Characteristic of Study Participants

One questionnaire was incomplete, hence a total of 403 participants were included in the final analysis. Out of 403 AGYW, 243 (60%) were from Temeke District. The majority of AGYW were in the age group between 21 and 24 years old. Most of the AGYW attained a primary level of education (59.6%) and their primary occupations were sex work or owned small businesses. Most (55.6%) AGYW reported to have no children and about one third (31.5%) were either single or had partner but did not cohabitate with them. The majority of AGYW had an estimated income of less than TZS.25,000 (~USD 11) per day (Table 1). 

### 3.2. Knowledge of Gender-Based Violence Definition among AGYW

Table 2 describes the levels of knowledge on gender-based violence among AGYW. Out of 403 AGYW, about 8 out of 10 (77.9%) participants reported to have a moderate and good levels of knowledge in defining GBV. A majority (79.9%) of participants correctly identified sexual violence as a form of gender-based violence. Even though sexual violence was the most frequently mentioned form of violence in both quantitative and qualitative study elements, a majority of participants reported that this was largely accepted by their communities because of their religious beliefs and community expectations for women to be submissive to their partners. However, when violence progressed to more severe levels, for example being forced to engage in anal sex was often described by participants as intolerable. 

Physical violence was viewed by many participants as a normal behavior between partners. Women expressed that, at times they expected and accepted physical violence due to its frequent occurrence. Furthermore, emotional violence was reported as the least tolerable form of violence among women.

“My partner calls me names and abuses me all the time. He makes all of the decision when it comes to how we use all the money. I think with this, I am already used to it but am scared when he beats me”

(AGYW, 19)

Despite the high levels of violence, some participants reported that no form of violence was neither acceptable nor tolerable, even between intimate partners. This clearly reflects a progressive thinking among AGYW towards community tolerability of GBV.

Similarly, qualitative results showed that participants were knowledgeable about what constitutes GBV. Some of the participants understood the typology of GBV, particularly sexual and physical violence to including acts of pushing, slapping, kicking, knocking, hitting, throwing objects and violent sexual behaviors. Participants often described the general concepts of gender-based violence using real examples of their own lived experiences. Most participants also described what constitutes gender-based violence using five Kiswahili terms. The term “fujo” (chaos and disorder), “kupigwa” (physical violence), “kudhalilishwa” (insults/name calling), “ugomvi” (quarrel) and “kubakwa” (rape).

Despite participant’s knowledge about GBV and its typology, participants described that there is a tendency to either justify, normalize, or accept acts of violence among AGYW.

“GBV… it is something normal and usually happens to most of us. I know many girls who have experienced GBV including myself who has been raped and beaten. I was burnt by a cigarette when I refused a man’s advancement [proceeded to show interviewer healed burn wound]”

(AGYW, 01)

“Sometimes women can be stubborn and a man can beat you to correct you, you know how women can be sometimes [chuckling] but when the beating becomes too much and without any good reason then it becomes a problem”

(AGYW, 04)

Some participants also suggested that men should be involved in future GBV interventions to increase their knowledge to mitigate violence perpetration.

“Many of us [AGYW] in this group understand what GBV is, but the problem is with men. They are the ones who beat and rape us. I think it would be good if men are involved in such [JHPIEGO SAUTI] projects and not only women”

(AGYW 12)

### 3.3. Perceptions of and Experiences with GBV Health Services 

Table 3 demonstrates perceptions of and experiences with GBV health services among AGYW. On average, about 31% of the participants strongly agreed or agreed with the following statements about available GBV health services. Most of them (47.6%, n = 192) disagreed with the statements that HCWs can prevent GBV reoccurrence. The least reported and known GBV health service among participants was the collection of forensic evidence (10.7%, n = 43) which includes collection of semen, saliva, clothing fibers, hair and blood samples. About half of participants (50.4%, n = 203) agreed that health care facilities could link them to legal bodies (Table 3).

The qualitative results mirrored the quantitative Likert scale results regarding perceptions of and experiences with GBV health services among AGYW. Some participants mentioned that they were aware of the availability of HIV screening services for GBV survivors at health facilities. However, others were not aware about the process of receiving HIV prevention services, specifically post-exposure prophylaxis (PEP) following sexual assault. Similarly, some participants were aware of provision of pregnancy tests at health facilities but were not aware that emergency contraceptive services available to prevent pregnancy.

“Haaa…it can never be…how can one remove the HIV virus from your blood after being raped by someone who has the virus…it is impossible. Likewise, how can one stop pregnancy when you have been raped and conceived. Unless one chooses to abort. I don’t think this is ever possible”

(AGYW 09)

Some participants mentioned that they were aware of linkages to legal aid services such as the police GBV division from the health care facilities, but they were afraid to go to the police station for fear of name calling.

“When I was once violated, I went to the police to report it for further action, however the police were not supportive and even started asking me what I was doing late at night with men. They even started condemning me that I was prostituting. I would rather not go back to them.”

(AGYW 03)

Qualitative findings also indicated that some participants were not aware of psychological and social services available to GBV survivors.

“As I told you I was raped when I went to the club on my form four graduation day [approximately 18 years old] where I ended up being raped by four strangers. My mother took me to a health facility where I was treated, and the doctor told me to forgive and forget what was done to me. How do I just forgive and forget? [starts crying angrily] And no, I was not linked to any social worker, how are they supposed to help?”

(AGYW 07)

### 3.4. Access to Gender-Based Violence Services among AGYW

**Figure 2:**
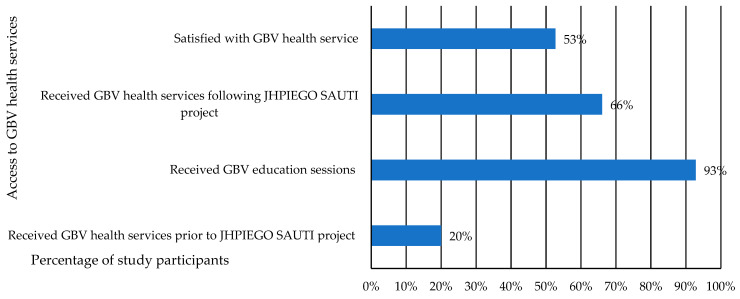
Access to GBV health services pre-and post- JHPIEGO SAUTI project implementation.

As illustrated in Figure 2, of all 403 participants in our study who had experienced lifetime gender-based violence (as per the study inclusion criteria), only about 20% had accessed GBV health services prior to joining the JHPIEGO SAUTI project. Most participants (about 93%), reported having received the JHPIEGO SUATI social behavior change and communication education (SBCC) program which entailed education on GBV, HIV, family planning and drug abuse prevention and provided escorted referrals to GBV health facilities. However, only 66% agreed to access GBV health services even after having received the education program. Overall, very few participants utilized GBV services prior to the JHPIEGO SAUTI project but after enrolment into the project, the number of participants utilizing GBV escorted referral services increased by 46%.

Further, Table 4 expands on the reasons for refusing GBV escorted referrals following the educational intervention. About three quarters (73.7) of the participants cited fear of HIV testing, confirming an HIV positive results or not having physical health issues as the main reasons for refusing escorted referrals to GBV services, thereby challenging successful linkage to GBV services.

In the qualitative element of this study, most participants additionally identified that time was not an important factor to accessing immediate GBV health services following GBV if they eventually sought help when deemed necessary by the participant. For our study, *time* was defined as 0–72 h following violence.

“When I was raped, I decided to keep quiet, I did not even tell my mother I was only 15 years old. I started to bleed [vaginal bleeding] but still kept quiet hoping it would somehow stop. After a week I started having painful abdominal pain and bad smell from my private area. I had to tell my mother who eventually took me to the hospital. The doctor blamed me for not reporting [the incident] sooner, so not much could be done. He gave me several pills and I got better eventually. That incident has affected me so badly. I still hate men, I am 23 years now and don’t wish to be with a man again.”

(AGYW 17)

Some participants who visited health facilities also reported that they were informed by HCWs that it was difficult to obtain forensic evidence for AGYW who were late to health facilities, especially for victims of sexual violence.

“After joining this [JHPIEGO SAUTI] project, they advised me to go the hospital because I was raped once. The doctor at the hospital informed me if it happens again, I should rush to the hospital immediately so that I can get pills to prevent from HIV and pregnancy and also, to get evidence for the police”

(AGYW 15)

## 4. Discussion

The findings of this study provided important insights into the GBV health service response among adolescent girls and young women in Tanzania. In summary, the majority of AGYW had relatively good GBV knowledge regarding its definition and forms of violence. However, AGYW had poor perceptions of and experiences with GBV health services, particularly surrounding information related to emergency contraceptives and post-exposure prophylaxis (PEP) as well as the availability of psychological and referral services (e.g., social workers and legal bodies). Even though AGYW had good GBV knowledge, the majority did not access the GBV services and even fewer were satisfied with the health services they had received. 

### 4.1. Knowledge of GBV among AGYW

AGYW maintained relatively good knowledge of GBV with regards to its definition and knowledge of forms of violence experienced by Tanzanian AGYW. Relatively good understanding of GBV may be attributed to the fact that our study participants were enrolled into an ongoing project that provided GBV education (i.e., SBCC education). Additionally, GBV knowledge can also be attributed to ongoing GBV interventions offered by the government initiatives, international development partners and non-governmental organization. Knowledge of GBV among AGYW was similar to findings from another study conducted in three regions of Tanzania (Mbeya, Dar-es-Salaam and Iringa) where participants maintained relatively high awareness (78.4%) of what constitutes GBV and were able to identify a range of violent behaviors including physical aggression, insults, beatings, being threatening with an object and the intended destruction of property [36]. However, in our study, not all respondents had sufficient knowledge of GBV despite enrollment in the JHPIEGO SAUTI project, thereby indicating a need for a more comprehensive educational intervention at community level. Future interventions should aim not only to mitigate GBV risk associated with high-risk social behaviors (e.g., engaging in concurrent sexual relationships, drugs and alcohol misuse) but should also aim to promote health and wellness.

Physical and sexual violence were the most frequent forms of violence reported by AGYW who often stated forms of violence that they themselves had experienced. Surveys administered in other African countries and around the globe similarly revealed that participants often only reported violence which they had encountered themselves [37]. This information is important to understand what GBV survivors perceive as violence and how they can be reached with sensitive care and support. Further, although the AGYW interviewed in this study had good knowledge about what constitutes GBV; there was often acceptance of GBV, likely perpetuated by cultural norms and gender roles which need to be addressed to increase uptake and continuation of GBV health services. According to United Nations Economic Commission for Africa and the African Centre for Gender and Social Development (2010), there are numerous reasons why GBV may be accepted by young women including the predominance of a patriarchal system, acceptance of GBV as a cultural norm or stigma attached to being a female victim of violence [38]. Interestingly, some cultural and social norms may perpetrate specific forms of violence. The participants in our study expressed that men have a right to control or discipline women through physical means and women’s acceptance of this thereby made them vulnerable to continued violence. Our study indicated that cultural and social norms value men as superior and more powerful than women. These norms and cultures subordinate women in many life’s spheres, from economic independence to decision-making power [39]. This often happens in contexts where societal norms allow the use of GBV to reprimand women and where men are expected to have the final say as a means to control women [40]. Our findings mirror that of studies conducted in Nigeria [40,41] where women described that cultural norms encouraged them to tolerate and accept acts of violence perpetrated against them. A further study conducted in South Africa by Safer-Spaces even attributed high rates of rape to the South African patriarchy [42]. These prevailing cultural gender norms may additionally give insight as to why physical and sexual violence in intimate relationships (e.g., domestic rape) are still considered culturally acceptable in Tanzania [43]. Additionally, marital rape is not recognized by Tanzanian law thereby preventing married women from seeking help and from obtaining appropriate services following sexual violence [44]. However, some AGYW in our study held a different view- that no form of violence is acceptable and tolerable even among intimate partners. Thus, these girls could function as peer champions to encourage other AGYW to access GBV services. Emotional and economic violence were the least frequent forms of violence reported by AGYW in our study. Experience with name calling yelling, threats, and financial control were of less concern to the participants than physical violence. This may be attributed by the fact that men always want to be seen in control of everything. In addition, women do not often talk for fear of the aggressors; threat against themselves and their children or relatives. However, other studies have reported emotional and economic violence may have a substantial impact on women’s mental wellbeing [45,46]. Thus, it is important for future interventions to focus on all forms of violence.

The gendered nature of violence against women in Tanzania are attributed in part to the patriarchal social system, norms of masculinity linked to male dominance, laws granting men control over women’s behaviors, attitudes that accept male violence as a way of resolving conflict and an inadequate provision of policies and infrastructure to successfully address GBV [47,48]. Additionally, some AGYW in our study mentioned that men were the main perpetrators of GBV and hence male involvement in GBV education is of vital importance. This finding is similar to other research conducted by Fleming et al. (2015) that echoed men as the main perpetrators of violence particularly in communities where power is concentrated in the hands of the male partner [43]. In the household level, GBV may be used to legitimize the dominant position of men while at the societal level, cultural norms allow men to use violence to maintain control. Despite the discriminatory and suppressive culture towards adolescent girls, gender attitudes and norms amongst AGYW that justify violence against them are beginning to change in Tanzania.

### 4.2. Perceptions of and Experiences with GBV Health Services among AGYW

Participants in this study were part of a national project that expanded coverage of a peer-based HIV and GBV prevention intervention for AGYW. However, AGYW had poor perceptions of and experiences with GBV health services such as emergency contraception and post-exposure prophylaxis, as well as psychological and referral services (i.e., social workers and legal bodies) which can negatively influence the utilization of future GBV services [14,49,50].These can have severe consequences on the health of GBV survivors including psychological suffering (e.g., anxiety, depression) and poor physical outcomes (e.g., injuries, shock and infectious diseases) [51]. These findings are similar to those found in a WHO multi-country study on women’s health and violence where perception of the availability and accessibility of HIV and GBV services for GBV survivors was on average 47% [52]. Additionally, our qualitative results indicated that AGYW were aware of the availability of HIV screening services following rape but were unaware of post-exposure prophylaxis for HIV prevention. This unique finding highlights a gap in GBV service awareness and should be considered by stakeholders to develop more comprehensive HIV and GBV education. 

Other studies have indicated that access to GBV health services, among women who reside in remote settings and have highlighted the relevance of socio-economic factors in knowledge and uptake of reproductive and child health services including GBV health services [53,54].

### 4.3. Access to Gender-Based Violence Health Services among AGYW

Social Behavior Change Communication (SBCC) education was provided to the AGYWs through the JHPIEGO SAUTI project with the aim of developing communication strategies to promote positive behaviors and empower women regarding access to HIV and GBV services. Our study (both quantitative and qualitative data) indicated however that many AGYW still declined GBV health services following the provision of SBCC education and escorted referrals. The majority of participants reported fear around HIV testing and confirming HIV positive results as primary reasons for declining escorted referrals. Further, AGYW often declined escorted referrals by their peers when they felt physically healthy. This meant a failure to seek medical support which may result in missed opportunities for proper diagnoses and treatment of underlying conditions associated with GBV. Thus, GBV education is insufficient and the government as well as other stakeholders should seek innovative models for enrolling women into care. Similarly, other studies mirrored our findings and confirmed that adolescent girls and women were afraid of testing and knowing their HIV status, consequently causing underutilization of health services [55,56,57]. Access to GBV care, is an essential element in achieving quality of life for AGYW, hence it is important for policy makers and project implementers to educate AGYW about the available GBV health services to improve access to GBV health services. Based on our findings, the empowerment and facilitation of GBV health service access for AGYW is vital for treating the consequences of GBV and preventing future instances of GBV. Measures such as comprehensive information distribution regarding the availability of GBV health service provision at health facilities and other referral services is vital to increase the uptake of GBV services. Additionally, health care providers should educate AGYW about the importance of seeking GBV services immediately following violence (0–72 h following an instance of violence) to increase efficacy of preventative (e.g., HIV and pregnancy prevention) and forensic services (e.g., body fluid collection). Health care providers should also link GBV survivors to psychosocial support in order to provide early mental health intervention and to provide a safety plan to prevent future violence. For instance, the JHPIEGO SAUTI project was involved in implementing income-generating activities with the aim of economically empowering AGYW. This approach speaks to the findings of a previous systematic review in low-and-middle-income countries which found out that higher education and household economic factors are critical in determining GBV knowledge and type of services access [58].

Even though this analysis highlights valuable insights to GBV knowledge as well as perceptions of and experiences with GBV health services, the study was not without limitations. As a retrospective cross-sectional study, the study had an inherent weakness of recall bias, however all efforts were made to ensure the effect of the recall bias was minimal. Furthermore, study participants were sampled from an ongoing GBV intervention (i.e., JHPIEGO SAUTI) which may have an influenced their knowledge of GBV services. It should be noted however that participants were randomly selected from the population to be included in the JHPIEGO SAUTI project, thereby presenting a representative sample of urban AGYW in Tanzania.

## 5. Conclusions

The study indicates that—despite fairly good knowledge of GBV—the knowledge of the specific roles and content of GBV health services was limited and accessibility and the use of GBV health services was low. Yet the study was conducted in urban Dar es Salaam where services are expected to be accessible. Major barriers were posed by the participants including fear of HIV testing, confirmation of a HIV—positive result and stigma associated with HIV treatment. Thus, a key challenge to accessing GBV health services is the user-friendliness and quality of care surrounding HIV prevention and HIV treatment. In this context, one-stop health care centers are possible facilitators to provide comprehensive GBV services (including HIV services) because all services can be provided under one roof.

Health systems should foremost promote and train health care workers on client-provider interactions to improve health system responsiveness and avoid stigma in the healthcare settings, thus, improving the health care experience. Furthermore, continued education about GBV and GBV health services should be required to dispel stigma and discrimination among health care workers and to enhance participation in care and support activities. Additionally, civil society organizations and program implementers should increase uptake of GBV health activities at the community level to strengthen referral and linkage to care for GBV survivors.

Finally, many AGYW are exposed to and live in unsafe environments hence broader efforts such as community sensitization campaigns via community stakeholders including local government authorities and religious leaders in partnership with the Ministry of Health can increase access to comprehensive GBV health services. Community sensitization programs should focus on addressing HIV and GBV health services to improve access to GBV health services and increase GBV health service experiences. The current study has potential to inform and contribute to the treatment guidelines for gender-based violence in Tanzania. Overall, our study aims to inform the design of future AGYW GBV interventions to subsequently reduce instances of HIV, sexually transmitted infections, pregnancy and future GBV thereby working towards universal access to sexual and reproductive health and reproductive rights as outlined in the SDG 5.

## Figures and Tables

**Table 1:** Socio-demographic characteristics of participants (n = 403).

Characteristics	Frequency n (%)
District Name	
Kinondoni	161 (39.7)
Temeke	242 (60.3)
Age	
15–17	44 (10.9)
18–20	129 (32.0)
21–24	230 (57.1)
Level of Education	
No Formal Education	97 (24.1)
Primary Education	240 (59.6)
Secondary and Higher Education	66 (16.4)
Primary Occupation	
Employed	48 (11.9)
Owned Small Business	83 (20.6)
Student	12 (3.0)
Sex Worker	151 (37.5)
Unemployed	109 (27.1)
Number of children	
None	224 (55.6)
One	135 (33.5)
Two and Above	44 (10.6)
Marital Status	
Single	127 (31.5)
Married	97 (24.1)
Cohabiting	29 (7.2)
Separated/Divorced	30 (7.4)
Have a Partner but not Cohabitating	120 (29.8)
Estimated Income (TZS.)	
0–25,000	384 (95.3)
25,001–50,000	11 (2.8)
50,001 and Above	8 (2.0)

1 USD = 2300 TZS.

**Table 2:** Knowledge of gender-based violence definition among AGYW.

	Level of Knowledge		
	Good	Moderate	Limited
Ability to Define GBV	108 (26.8%)	206 (51.1%)	89 (22.1)
	Yes (%)	No (%)	
Forms of Violence			
Sexual Violence	322 (79.9%)	81 (20.1%)	
Physical Violence	262 (65.0%)	141 (35.0%)	
Emotional Violence	166 (41.2%)	237 (58.8%)	
Economic Violence	107 (26.6%)	296 (73.4%)	

Values are expressed as number (percentage).

**Table 3:** Perceptions of and experiences with GBV health services among AGYW.

Statement	Strongly Agree/Agree	Neutral	Disagree/Strongly Disagree
1. HCWs can Prevent Violence from Reoccurring	117 (29.1%)	94 (23.3%)	192 (47.6%)
2. HCWs offer PEP for the Prevention of HIV Following Rape	146 (36.2%)	78 (19.4%)	179 (44.4%)
3. HCWs offer EC for Prevention of Pregnancy Following Rape	82 (20.3%)	87 (21.6%)	234 (58.1%)
4. HCWs offer Psychological and Counselling Services to GBV Survivors	87 (21.6%)	109 (27.0%)	207 (51.4%)
5. HCWs can Link GBV Survivors to Psychosocial Support (Counselling)	59 (14.6%)	102 (25.3%)	242 (60.1%)
6. HCWs can Link GBV Survivors to Legal Aid Services	203 (50.4%)	84 (20.8%)	116 (28.8%)
7. HCWs can Assist in Collection of Evidence for Forensic Medical Services	43 (10.7%)	95 (23.8%)	264 (65.5%)
8. GBV Survivors can Access GBV Health Services for the Treatment of Physical Injuries	259 (64.3%)	62 (15.4%)	82 (20.3%)

Values are expressed as n (%); HCWs—Health care workers; PEP—Post-Exposure Prophylaxis; EC—Emergency Contraception.

**Table 4:** Reasons for refusing escorted referral to GBV services following SBCC training.

Main Reason for Refusing a GBV Escorted Referral	n = 155	%
Fear of HIV Testing	49	31.4
Fear of Confirming an HIV-Positive Status	29	18.6
GBV Occurred a Long Time Ago	21	14.1
Accessed Health Facility Prior to JHPIEGO SAUTI Project	19	12.2
Felt physically healthy	37	23.7

Values are expressed as n (%); SBCC—sexual and behavior changes communication; JHPIEGO—Johns Hopkins Program for International Education in Gynecology and Obstetrics.

## Data Availability

The full dataset (transcripts) generated and analyzed during the study are not publicly available due to privacy concerns because of the nature of the study. Data can be made available by reasonable request through the corresponding author.
